# PCSK9 expression and cancer survival: a prognostic biomarker at the intersection of oncology and geroscience

**DOI:** 10.1007/s11357-025-01733-3

**Published:** 2025-06-27

**Authors:** Zoltan Ungvari, Otília Menyhart, Andrea Lehoczki, Monika Fekete, Giampaolo Bianchini, Balázs Győrffy

**Affiliations:** 1 Vascular Cognitive Impairment, Neurodegeneration and Healthy Brain Aging Program, Department of Neurosurgery, University of Oklahoma Health Sciences Center, Oklahoma City, OK USA; 2 Stephenson Cancer Center, University of Oklahoma, Oklahoma City, OK USA; 3 Oklahoma Center for Geroscience and Healthy Brain Aging, University of Oklahoma Health Sciences Center, Oklahoma City, OK USA; 4 Department of Health Promotion Sciences, College of Public Health, University of Oklahoma Health Sciences Center, Oklahoma City, OK USA; 5 International Training Program in Geroscience, Doctoral College/Institute of Preventive Medicine and Public Health, Semmelweis University, Budapest, Hungary; 6 Cancer Biomarker Research Group, Institute of Molecular Life Sciences, Hungarian Research Network, Magyar Tudósok Körútja 2, 1117 Budapest, Hungary; 7 National Laboratory for Drug Research and Development, Magyar Tudósok Körútja. 2. 1117, Budapest, Hungary; 8 Department of Bioinformatics, Semmelweis University, 1094 Budapest, Hungary; 9 Doctoral College, Health Sciences Division, Semmelweis University, Budapest, Hungary; 10 Institute of Preventive Medicine and Public Health, Semmelweis University, Budapest, Hungary; 11 Fodor Center for Prevention and Healthy Aging, Semmelweis University, Budapest, Hungary; 12 Department of Medical Oncology, IRCCS Ospedale San Raffaele, Milan, Italy; 13 Vita-Salute San Raffaele University, Milan, Italy; 14 Department of Biophysics, Medical School, University of Pecs, Pecs, 7624 Hungary

**Keywords:** PCSK9, Cancer survival, Prognosis, Tumor microenvironment, Immune modulation, Aging, Metastasis, Lipid metabolism, Breast cancer, Immunotherapy, Biomarkers, Lifestyle factors, Cholesterol metabolism, Vascular aging, Oncology, Tumor progression

## Abstract

Proprotein convertase subtilisin/kexin type 9 (PCSK9) is primarily recognized for its role in cholesterol metabolism; however, emerging evidence suggests it plays a broader role in the regulation of cellular aging mechanisms and the pathogenesis of age-related diseases. Given that cancer is an age-related disease, PCSK9 has garnered attention for its potential impact on tumor progression and patient survival. In this study, we conducted a comprehensive analysis of PCSK9 expression across multiple tumor types, assessing its prognostic significance using RNA sequencing data from The Cancer Genome Atlas (TCGA) and gene expression microarray data from the Gene Expression Omnibus (GEO). Cox proportional hazards regression models and Kaplan–Meier survival analyses were employed to evaluate overall survival (OS) associations. Our findings reveal that elevated PCSK9 expression is associated with improved OS in breast and ovarian cancers, particularly in Luminal B breast cancer subtypes. Conversely, high PCSK9 expression correlates with worse OS in bladder cancer, renal clear cell carcinoma, melanoma, and pancreatic cancer. Notably, while PCSK9 expression is significantly upregulated in melanoma and bladder tumors, it is downregulated in renal clear cell carcinoma, yet relatively higher expression among renal tumors still predicts poorer survival. No significant associations between PCSK9 expression and OS were observed in colon, liver, gastric, lung, prostate, head and neck cancers, or low-grade gliomas in the available datasets.In conclusion, our study identifies PCSK9 as a prognostic biomarker with distinct, tumor-specific survival implications. Its dual role—associating with improved survival in some cancers while correlating with worse outcomes in others—suggests that PCSK9 may influence cancer progression through context-dependent mechanisms. Future research should focus on elucidating the mechanistic underpinnings of these associations and exploring the diagnostic and therapeutic potential of targeting PCSK9 in oncology.

## Introduction

Cancer is a leading cause of morbidity and mortality worldwide, with incidence rates rising significantly with age. According to the geroscience concept, cancer progression is influenced by molecular and cellular mechanisms that also underlie aging [1], including genomic instability, chronic inflammation, cellular senescence, and metabolic dysregulation. Applying geroscience principles to cancer research has led to the identification of several aging-related pathways as key modulators of cancer pathogenesis, unveiling novel therapeutic targets and strategies.

A growing body of evidence suggests that proprotein convertase subtilisin/kexin type 9 (PCSK9), a protein best known for its role in cholesterol metabolism, plays a crucial role in the regulation of cellular and molecular processes associated with aging and contributes to the pathogenesis of various age-related diseases [2–4]. PCSK9 is a secreted serine protease that regulates low-density lipoprotein receptor (LDLR) degradation, thereby controlling circulating cholesterol levels [2, 5]. While PCSK9 inhibition has been widely explored as a therapeutic strategy for cardiovascular diseases [6], recent studies indicate that its functions extend beyond lipid metabolism [5, 7]. Dysregulated PCSK9 expression has been linked to multiple age-associated conditions, including cardiovascular dysfunction [8], liver aging [9], and metabolic syndrome. These findings raise intriguing questions about PCSK9’s potential role in cancer, another major age-related disease [2, 3, 10].

Recent studies have implicated PCSK9 in multiple aspects of cancer progression, including tumor cell proliferation, immune evasion, and metastasis [2, 10–13]. Elevated PCSK9 expression has been observed in several malignancies, including pancreatic adenocarcinoma [14] and esophageal squamous cell carcinoma, where higher levels correlate with advanced clinical stage, lymph node metastasis, and poor overall survival. Additionally, PCSK9 has been shown to modulate immune cell infiltration within the tumor microenvironment, with potential implications for cancer immunotherapy [3, 10, 15, 16]. Inhibition of PCSK9 can enhance major histocompatibility complex class I (MHC-I) expression on tumor cells, increasing their recognition by cytotoxic T cells and improving immune-mediated tumor clearance [2, 5, 17]. Despite these emerging insights, the prognostic significance of PCSK9 expression across different cancer types remains poorly understood. Some studies suggest that high PCSK9 expression is associated with worse survival outcomes in certain malignancies, while others report a protective effect [18–25]. Given these conflicting findings, a comprehensive pan-cancer analysis of PCSK9 expression and survival outcomes is needed to clarify its role in oncology.

In this study, we conduct a comprehensive analysis of PCSK9 expression across multiple cancer types using large-scale transcriptomic datasets. By evaluating its prognostic significance through associations with overall survival in diverse tumor cohorts, we aim to elucidate the context-dependent role of PCSK9 in tumor biology. Our findings contribute to a deeper understanding of PCSK9 as both a potential prognostic biomarker and a therapeutic target in oncology.

## Methods

### RNA-seq database

We retrieved RNA sequencing data from The Cancer Genome Atlas (TCGA), which contains comprehensive transcriptomic profiles and clinical follow-up for a large cohort of patients. Only tumor types with more than 100 cancer specimens were included to ensure a sufficiently robust sample size for each analysis. The basis for expression analyses was the RNA-seq HTSeq count data generated by the Illumina HiSeq 2000 RNA Sequencing Version 2 platform. The DESeq package, which uses a negative binomial distribution, was utilized to normalize raw counts [26]. Gene annotation was carried out using the Bioconductor “AnnotationDbi” package (http://bioconductor.org/packages/AnnotationDbi/) to map Ensembl transcript IDs to gene symbols (*n* = 25,228). A second scaling normalization was then performed, setting the mean expression of all genes in each sample to 1000 to minimize batch effects.

### Gene array database

To build a comprehensive dataset of gene expression profiles with associated survival information, we also retrieved data on solid tumors from the Gene Expression Omnibus (GEO; http://www.ncbi.nlm.nih.gov/geo/). Studies were included if they met three criteria: (1) raw data files were available, (2) documented overall survival (OS) outcomes with censoring data were provided, and (3) at least 30 patients were enrolled. We focused on Affymetrix HG-U133A (GPL96) and HG-U133 Plus 2.0 (GPL570) microarray platforms due to their wide usage and the overlap of 22,277 probe sets. Aligning closely related microarray platforms is crucial because different gene-expression technologies can vary in measurement accuracy and dynamic range. All gene expression arrays underwent multi-step preprocessing to ensure data consistency and reliability. Each array was independently normalized using the MAS5 algorithm, whose performance closely aligns with RT-PCR reference values [27]. Because MAS5 operates on individual samples, adding or removing arrays does not compromise overall dataset integrity. Next, we applied a global scaling step to align the mean expression of the 22,277 overlapping probes at a fixed value of 1000, effectively reducing batch effects and allowing for seamless comparisons across different datasets. Only the probes available in the GPL96 platform were retained to avoid platform-specific biases [28]. The JetSet algorithm was utilized to identify the highest-quality probe set for each gene. Samples displaying identical expression values across all samples were considered duplicates; only the first occurrence was retained. Additional quality checks involved assessing background signal intensity, noise levels, and the proportion of present calls, all of which gauge signal fidelity. Experimental consistency was verified by monitoring bioBCD spikes, and RNA integrity was confirmed by examining the 3′/5′ ratios of housekeeping genes GAPDH and ACTB. Only samples that satisfied all predefined quality thresholds and fell within the 95% confidence interval for continuous variables were retained, whereas those failing any criterion were designated as outliers and excluded. Through this comprehensive preprocessing and quality control framework, the dataset achieved a high accuracy and consistency suitable for downstream analyses.

### Survival analysis

A Cox proportional hazards regression was performed to explore the relationship between PCSK9 expression and OS using the “survival” R package (v2.38; http://CRAN.R-project.org/package=survival/). We calculated log-rank p-values, hazard ratios (HR), and 95% confidence intervals (CI) and plotted Kaplan–Meier curves to visualize survival differences.

We iterated through all possible expression cutoffs between the lower and upper quartiles to avoid using a single arbitrary cutoff (e.g., the median). Each cutoff was tested in a separate Cox regression. Multiple hypothesis testing was controlled by calculating the false discovery rate (FDR), with significant findings defined as FDR ≤ 20%. The best-performing cutoff (lowest p-value) was used to generate final Kaplan–Meier curves.

For the selected cancer types (breast, colon, gastric, lung, ovarian, and pancreatic cancer), both gene array and RNA-seq transcriptomic data and the corresponding clinical outcome measures were available. We performed the survival analysis in both datasets separately and reported the results for each cancer type. Findings were considered significant at concurrent p < 0.05 and FDR ≤ 20% values.

### Gene expression differences

Finally, we assessed the differences in PCSK9 expression between normal and tumor samples by utilizing the TNM plotter webserver (https://tnmplot.com) [29]. The TNM plotter incorporates data from public repositories and allows interactive visualizations to identify tumor-specific gene dysregulation.

## Results

### Database construction

The RNA-seq database includes samples from 14 solid tumor types: bladder, breast, colorectal, gastric, head and neck, lung, ovarian, pancreatic, prostate and liver cancer, glioblastoma multiforme, low-grade glioma, melanoma, and renal cell carcinoma. For breast cancer, additional samples with RNA-seq and clinical outcome data were available in the GSE96058 dataset, which was included in our analyses. Clinical data of patients with available PCSK9 expression can be found in Tbale 1AB.

**Table 1:** Clinical attributes of tumor patients with available PCSK9 expression levels and data on overall survival in the RNA-seq databases

**A.**							
**Tumor type**	**Classification**		**n of patients**	**Tumor type**	**Classification**		**n of patients**
**Bladder (*n* = 403)**	*subtype*	papillary	130	**Liver (*n* = 364)**	*gender*	male	246
		non-papillary	268			female	118
	*stage*	1	1		*stage*	1	170
		2	129			2	83
		3	138			3	83
		4	132			4	4
	*grade*	low grade	20	**LGG (*n* = 514)**	*gender*	male	283
		high grade	380			female	228
	*gender*	male	297		*histology*	astrocytoma	193
		female	106			oligodendroglioma	188
**Breast (*n* = 1065)**	*age*	< 50	293			oligoastrocytoma	130
		> 50	739		*grade*	2	246
	*subtype*	TNBC	207			3	264
		Luminal A	444	**Lung (*n* = 1001)**	*gender*	male	597
		Luminal B	348			female	399
		HER2 +	66		*histology*	adenocarcinoma	502
	*cytokeratin*	negative	454			squamous cell carcinoma	494
		positive	259		*stage*	1	515
	*T*	1	279			2	282
		2	617			3	163
		3	127			4	33
		4	36	**Melanoma (*n* = 460)**	*gender*	male	284
	*N*	negative	499			female	175
		positive	353		*stage*	1	77
	*M*	positive	22			2	139
		negative	886			3	169
**Colon (*n* = 463)**	*cohort*	colon	293			4	22
		rectal	159		*tumor site*	trunk	164
	*histology*	adenocarcinoma	252			extremities	191
		mucinous adenocarc	38			head and neck	48
	*stage*	1	75	**Ovarian (*n* = 373)**	*grade*	1	1
		2	163			2	42
		3	130			3	319
		4	65			4	1
	*gender*	male	246		*stage*	1	1
		female	206			2	21
**Gastric (*n* = 410)**	*gender*	male	255			3	291
		female	135			4	57
	*stage*	1	52	**Pancreas (*n* = 177)**	*gender*	male	96
		2	123			female	80
		3	168		*stage*	1	21
		4	39			2	145
	*grade*	1	10			3	3
		2	138			4	4
		3	233	**Prostate (*n* = 488)**	*histology*	acinar adenocarcinoma	472
**GBM (*n* = 152)**	*gender*	male	98			other adenocarcinoma	15
		female	54		*Gleason-score*	7 +	442
**Head and neck**	*gender*	male	366			8 +	197
**(*n* = 499)**		female	133			9 +	135
	*stage*	1	25	**Renal cell**	*gender*	male	345
		2	69	**carcinoma (*n* = 533)**		female	186
		3	78		*stage*	1	268
		4	259			2	57
						3	124
						4	84
**B.**							
**Tumor type**	**Classification**		**Number of patients**				
**Breast (*n* = 2976)**	subtypes	basal	308				
		Luminal A	1504				
		luminal B	668				
		HER2 +	295				
		positive	1067				
		negative	1820				

This table summarizes demographic and clinical variables across solid tumor cohorts included in the TCGA RNA-seq dataset (Panel A), and breast cancer patients in the GSE96058 dataset (Panel B). Variables include tumor type, histologic subtype, tumor stage, grade, gender, age, and lymph node status. Subtype classifications for breast cancer include TNBC (triple-negative breast cancer), Luminal A, Luminal B, and HER2-enriched tumors. LGG = low-grade glioma; GBM = glioblastoma multiforme; TNM = tumor, node, metastasis. Data reflect the number of patients in each category

The gene array database consists of six solid tumor types (breast, colon, gastric, lung, ovarian, and pancreatic cancer) with available PCSK9 expressions and OS outcome data. Clinical parameters of patients in the gene array dataset are illustrated in Table 2.

**Table 2:** Clinical characteristics of patients with available PCSK9 expression and overall survival data in the gene array datasets

Tumor type	Classification		Number of patients		Tumor type	Classification	Number of patients	
**Breast (*n* = 1880)**	*age*	< 50	370		**Lung (*n* = 2167)**	*gender*	male	819
		> 50	394				female	476
	*subtype*	basal	278			*histology*	adenocarcinoma	670
		Luminal A	377				squamous cell carcinoma	526
		Luminal B	177				large cell carcinoma	52
		HER2 +	111				large cell neuroendocrine	56
	*grade*	1	26			*stage*	T1	220
		2	64				T2	190
		3	204				T3	33
	*lymph node*	positive	230				T4	21
		negative	180		**Ovarian (*n* = 1657)**	*stage*	1	51
**Colon (*n* = 1061)**	*gender*	male	337				2	31
		female	402				3	421
	*stage*	1	74				4	61
		2	302			*histology*	endometrioid	21
		3	298				serous	517
		4	131			*grade*	1	41
	*grade*	1	17				2	159
		2	166				3	388
		3	30				4	18
**Gastric (*n* = 881)**	*gender*	male	347		**Pancreas (*n* = 87)**	*grade*	1	2
		female	187				2	32
	*stage*	1	62				3	29
		2	135				4	1
		3	197			*stage*	1	4
		4	139				2	44
							3	10
							4	6

This table presents patient-level clinical data for solid tumors analyzed using Affymetrix-based gene expression arrays. Included variables are age, gender, tumor grade, histologic subtype, cancer stage (TNM classification where available), and hormone receptor subtypes in breast cancer. Subtypes listed for breast cancer include Basal, Luminal A, Luminal B, and HER2 +. Histologic subtypes for lung, colon, gastric, pancreatic, and ovarian cancers are also included. The data represent the total number of patients within each category

### Cancer-specific prognostic value of PCSK9 expression

We evaluated PCSK9 expression across multiple cancer types to determine its prognostic utility. Below, we highlight the association between PCSK9 expression and survival outcome for each tumor category. Tumor types without significant associations are briefly noted. We also report differences in PCSK9 expression across normal and tumor samples based on the results generated by the TNM Plotter.

### Breast cancer

**Fig. 1:**
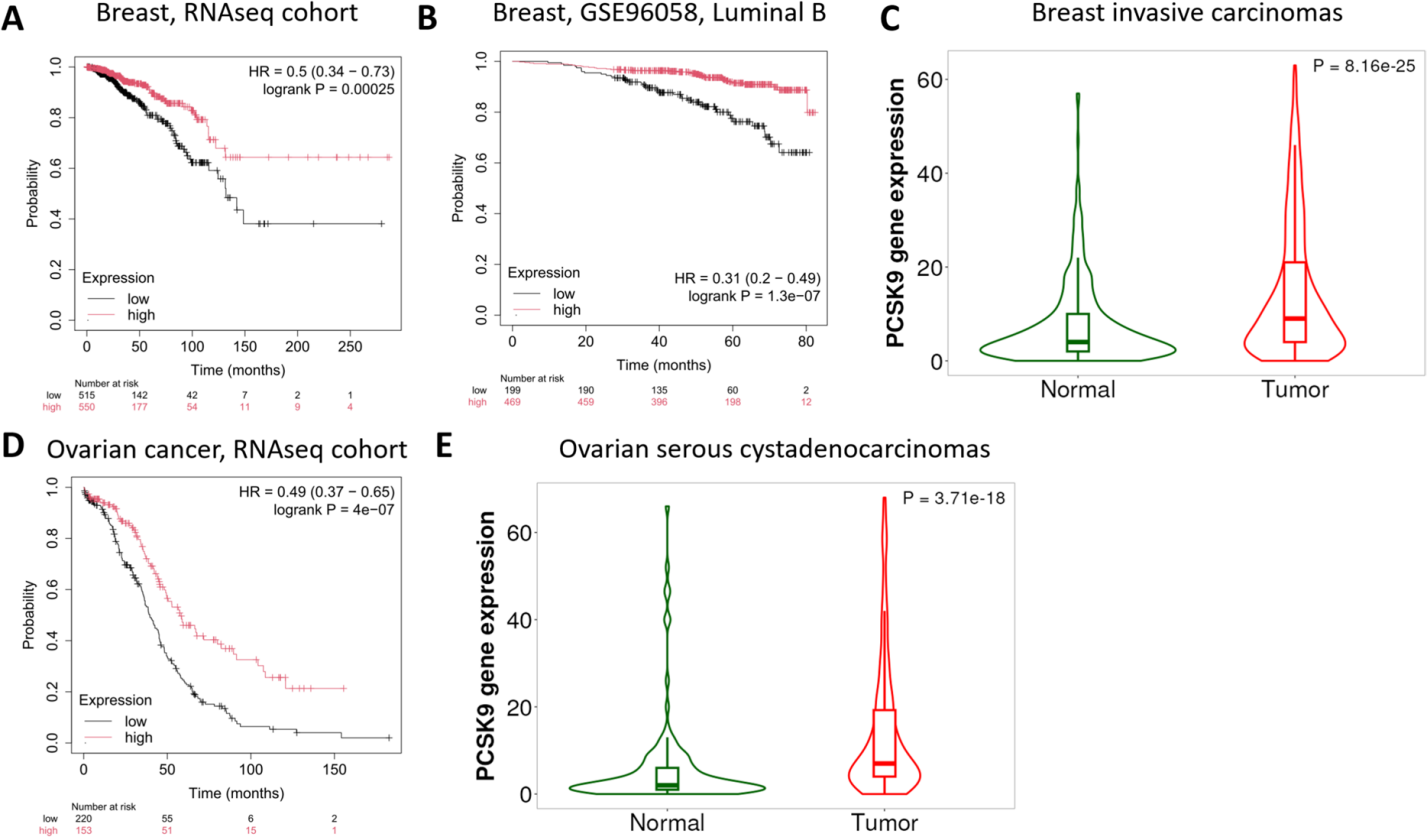
Association between PCSK9 expression and overall survival in breast and ovarian cancer.** A)** Kaplan–Meier curves illustrate the association between high expression of PCSK9 and improved OS in the full cohort of breast cancer patients in the RNA-seq dataset. **B)** Elevated expression of PCSK9 was associated with improved OS in the Luminal B subtype of GSE96058 cohort breast cancer patients. **C)** PCSK9 is significantly overexpressed in breast-invasive carcinoma compared to normal breast tissue samples. **D)** Increased expression of PCSK9 was associated with improved prognosis among ovarian cancer patients in the RNA-seq dataset. **E)** Investigating gene expression differences across ovarian serous cystadenocarcinomas and normal ovarian tissue revealed a significantly increased PCSK9 expression in the tumor samples. *HR, hazard ratio*

There was a significant correlation between high PCSK9 expression and improved OS in the RNA-seq breast cancer cohort (HR = 0.5, 95% CI = 0.34–0.73, p = 0.00025, FDR = 3%, Fig. 1A). Subtype-specific analyses showed similar trends, particularly in Luminal A (HR = 0.36, 95% CI = 0.21–0.64, p = 0.00028, FDR = 3%) and Luminal B tumors (HR = 0.24, 95% CI = 0.1–0.59, p = 0.00065, FDR = 5%), suggesting a potential subtype-dependent protective effect. The effect remained consistent across various clinical categories: stratification by age indicated that patients older than 50 with high PCSK9 expression had improved OS (HR = 0.45, 95% CI = 0.27–0.72, p = 0.00075, FDR = 5%). Additionally, analyses of TNM categories revealed significantly improved OS among T2 (HR = 0.4, 95% CI = 0.24–0.69, p = 0.00055, FDR = 5%), node-negative (HR = 0.3, 95% CI = 0.15–0.62, p = 0.00053, FDR = 5%), and metastasis-negative patients (HR = 0.51, 95% CI = 0.34–0.77, p = 0.001, FDR = 10%).

In the GSE96058 cohort, PCSK9 expression in the overall breast population did not reach significance, but a strong association emerged in Luminal B tumors (HR = 0.31, 95% CI = 0.2–0.49, p = 1.3e-07, FDR = 1%, Fig. 1B).

In contrast, in the gene array cohort, high PCSK9 expression did not show any significant association with OS in the whole population, nor did subtype-specific analyses (Basal, Luminal A, Luminal B, HER2-enriched) reveal a significant association with OS.

A comparison of normal and tumor tissues in the TNM plotter showed a statistically significant upregulation of PCSK9 in invasive breast carcinomas (p = 8.16e-25, Fig. 1C). However, subtype-specific data for breast cancer are not available within the TNM plotter. According to our analysis, PCSK9 expression confers benefits only within particular molecular subtypes; therefore, these findings do not necessarily represent a contradiction, as PCSK9 may fulfill context-dependent roles that vary across the stages of tumorigenesis, cellular environments, or treatment settings. Further mechanistic and functional analyses are needed to elucidate the molecular pathways by which elevated PCSK9 expression contributes to improved survival despite its overall upregulation in breast tumors.

### Ovarian cancer

In ovarian cancer, we found a strong association between high PCSK9 expression and improved OS in the RNA-seq dataset (HR = 0.49, 95% CI = 0.37–0.65, p = 4E-07, FDR = 1%, Fig. 1D). However, in the gene array cohort, the association only showed a weak trend in the same direction (p = 0.07).

The expression analysis across normal and tumor samples revealed significant upregulation of the PCSK9 gene in ovarian serous cystadenocarcinomas (p = 3.7E-18, Fig. 1E).

### Bladder cancer

**Fig. 2:**
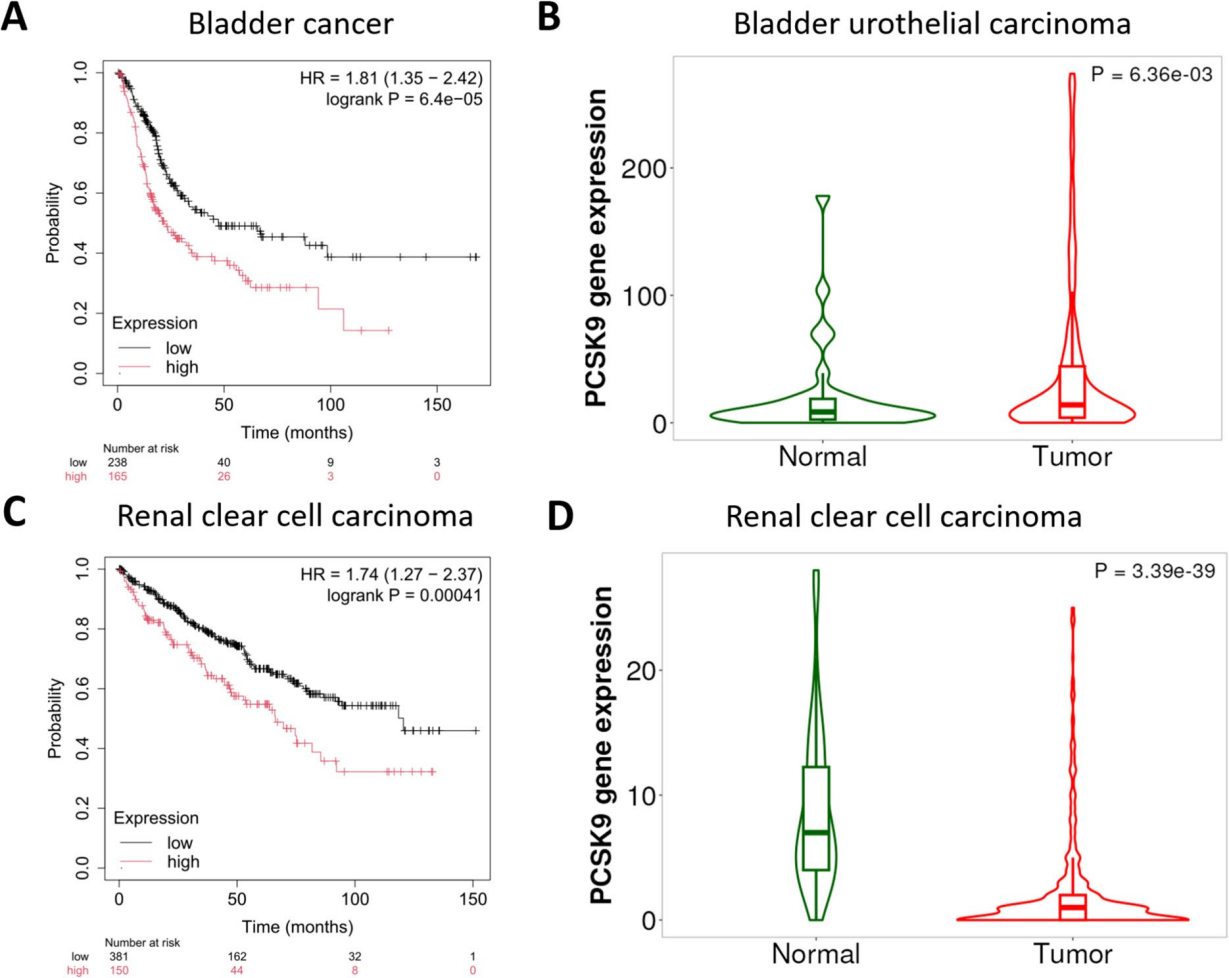
Association between PCSK9 expression and overall survival in bladder and renal clear cell carcinoma.** A)** Survival analysis in the RNA-seq bladder cancer cohort revealed an association between higher PCSK9 expression and worse overall survival. **B)** The violin plot comparing PCSK9 expression in normal versus bladder urothelial carcinoma tissues indicates elevated PCSK9 in tumor samples. **C)** Survival analysis in the RNA-seq renal clear cell carcinoma cohort shows worse overall survival for tumors with higher PCSK9 expression. **D)** Renal clear cell carcinoma tissues show reduced PCSK9 levels compared to normal kidneys despite poorer survival outcomes for cases with relatively elevated expression. *HR, hazard ratio*

In the RNA-seq bladder cancer dataset, high PCSK9 expression was associated with worse OS (HR = 1.81, 95% CI = 1.35–2.42, p = 6.4e-05, FDR = 1%, Fig. 2A). Subtype analyses indicated a significant effect in papillary bladder cancer (HR = 2.95, 95% CI = 1.6–5.47, p = 3e-04, FDR = 2%), and stage-specific analysis revealed a moderate association in stage 2 tumors (HR = 2.79, 95% CI = 1.34–5.8, p = 0.0041, FDR = 20%). High-grade tumors showed a stronger correlation (HR = 1.71, 95% CI = 1.27–2.3, p = 3e-04, FDR = 3%), and the association was also significant in male patients (HR = 2.15, 95% CI = 1.51–3.05, p = 1.2e-05, FDR = 1%).

Analysis with the TNM plotter revealed that bladder urothelial carcinoma tissues exhibit significantly higher PCSK9 expression than normal bladder tissue samples (p = 6.8E-03, Fig. 2B).

### Renal cell carcinoma

In the RNA-seq renal cell carcinoma cohort, high PCSK9 expression was significantly associated with worse OS (HR = 1.74, 95% CI = 1.27–2.37, p < 0.00041). Within the clear cell subtype, high PCSK9 expression conferred a higher mortality risk (HR = 2, 95% CI = 1.48–2.7, p = 3.5e-06, FDR = 1%, Fig. 2C), and in papillary carcinoma, we found a similarly detrimental association (HR = 2.56, 95% CI = 1.39–4.72, p = 0.0018, FDR = 20%).

Interestingly, PCSK9 expression is significantly downregulated in both clear cell (p = 3.4e-39) and papillary (p = 6.4e-36) renal carcinomas compared to normal kidney tissue (Fig. 2D**)**, suggesting an inverse expression pattern despite its prognostic value in these tumors. While tumors overall have reduced PCSK9 levels compared to normal kidneys, having a “high” level among the tumor group is still associated with a poorer prognosis.

### Melanoma

**Fig. 3:**
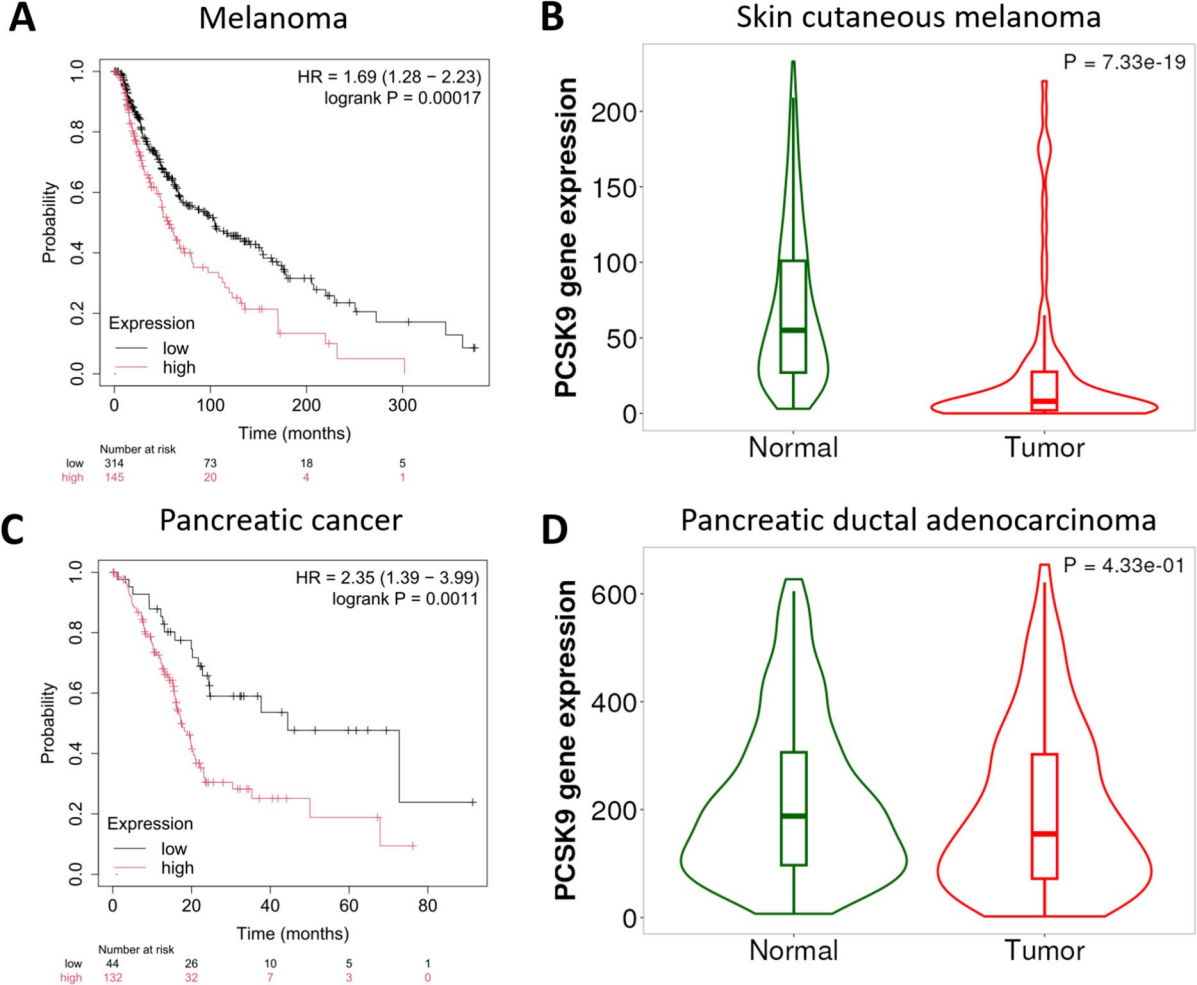
Association between PCSK9 expression and overall survival in melanoma and pancreatic cancer.** A)** Survival analysis in the RNA-seq melanoma cohort indicates that higher PCSK9 expression correlates with worse overall survival. **B)** Violin plot comparing PCSK9 expression in normal skin versus cutaneous melanoma tissues shows significantly elevated PCSK9 in tumors. **C)** Survival analysis in the RNA-seq pancreatic cancer cohort reveals that higher PCSK9 expression is linked to worse overall survival. **D)** PCSK9 expression in normal pancreas versus pancreatic ductal adenocarcinoma tissues is not significantly different. *HR, hazard ratio*

High PCSK9 expression in the RNA-seq melanoma cohort was associated with worse OS (HR = 1.69, 95% CI = 1.28–2.23, p = 0.00017, FDR = 2%, Fig. 3A), indicating a nearly 70% elevated mortality risk relative to lower expression levels. Skin cutaneous melanoma samples showed a significantly lower PCSK9 expression than normal skin tissue samples (p = 7.3E-19, Fig. 3B).

### Pancreatic cancer

In the RNA-seq pancreatic cancer dataset, high PCSK9 expression was associated with poor OS (HR = 2.35, 95% CI = 1.39–3.99, p = 0.0011, FDR = 10%, Fig. 3C), translating to more than twice the mortality risk. We did not find an association between PCSK9 and OS in the gene array dataset, but the sample size was substantially lower compared to the RNA-seq dataset (*n* = 87). Moreover, our analysis found no significant expression differences in PCSK9 in pancreatic ductal adenocarcinoma compared to normal pancreatic tissue samples (Fig. 3D**)**.

In summary, these findings demonstrate that PCSK9 expression may influence the clinical course of several cancers, either conferring a survival advantage, in particular breast and ovarium cancers, or correlating with worse OS in bladder, renal, melanoma, and pancreatic cancers. Further investigations are warranted to elucidate the biological mechanisms underlying these diverse effects.

### Lack of associations between PCSK9 expression and OS in several cancer types

No significant associations were observed in the gene array colon cancer cohort or the RNA-seq colon cancer dataset. However, PCSK9 expression was markedly higher in colon adenocarcinoma than in normal colon tissue (p = 1.61e-61), suggesting that although PCSK9 is overexpressed, it does not appear to influence OS in this cancer type.

Similarly, we found no associations between PCSK9 expression and OS in lung cancer, even when running histology-based subtype-specific analyses in either the gene array or the RNA-seq datasets. There was no association between PCSK9 expression and disease outcome in prostate cancer, head and neck, liver or gastric cancers, or low-grade gliomas in the available analyzed datasets.

## Discussion

Our study provides a comprehensive analysis of PCSK9 expression across multiple tumor types, highlighting its tumor-specific prognostic significance. We demonstrate that PCSK9 expression exhibits a complex, context-dependent relationship with overall survival, suggesting potential diverse roles in cancer progression and patient outcomes.

The differential prognostic implications of PCSK9 expression across cancer types [18–25] suggest that its role in tumor biology is likely influenced by factors such as tissue specificity [19], molecular subtype, and tumor microenvironment interactions. In breast and ovarian cancers, higher PCSK9 expression was associated with improved OS, particularly in Luminal B breast cancer subtypes [30], suggesting a potential tumor-suppressive or protective effect. Conversely, in bladder cancer, renal clear cell carcinoma, melanoma [31], and pancreatic cancer, elevated PCSK9 expression correlated with significantly worse OS, indicating a tumor-promoting role. The mechanisms underlying these divergent effects remain unclear [13], but may involve PCSK9-mediated regulation of tumor growth and apoptosis [32, 33], lipid metabolism [22], immune modulation [34–36], and angiogenesis.

Emerging evidence suggests that PCSK9 plays a role in modulating immune surveillance in tumors [10, 15, 18, 37]. PCSK9 is known to regulate MHC-I molecules, which are essential for immune recognition of tumor cells [17]. Inhibition of PCSK9 has been shown to enhance MHC-I expression, thereby increasing tumor cell visibility to cytotoxic T lymphocytes and enhancing immune-mediated tumor clearance [17, 38, 39]. The observed correlation between PCSK9 expression and immune cell infiltration, including CD8 + T cells and macrophages, further supports the notion that PCSK9 may influence antitumor immunity [17, 40]. The differential impact of PCSK9 expression on survival across cancers [38] could therefore be partially explained by variations in immune contexture and immune evasion strategies adopted by different tumors.

As cancer is an age-related disease, there is likely a connection between PCSK9, aging, and tumor progression. PCSK9 has been implicated in various aging-related pathophysiological processes [4, 41, 42], including vascular aging, metabolic dysregulation, oxidative stress [43, 44], senescence [45], and chronic inflammation that may indirectly influence cancer development and progression. In particular, PCSK9-driven alterations in lipid metabolism and inflammatory signaling pathways may contribute to the pro-tumorigenic environment observed in some cancers. Understanding the intersection of PCSK9, aging, and cancer could provide novel insights into shared pathogenic mechanisms and potential therapeutic targets.

A specific missense variant of PCSK9, rs562556 (V474I), has been identified as a key determinant of breast cancer metastasis [11, 12]. This gain-of-function mutation, prevalent in individuals of European ancestry, enhances PCSK9 activity and is associated with reduced survival in multiple breast cancer cohorts [11]. Mechanistic studies in genetically engineered mice confirmed that the variant promotes metastasis, while PCSK9 deletion suppresses it [11]. A key downstream target, low-density lipoprotein receptor-related protein 1 (LRP1), was found to mediate these effects [11]. PCSK9 downregulates LRP1, driving a pro-metastatic gene signature and enhancing tumor cell migration and invasion [11]. These findings suggest that targeting PCSK9 could be a potential strategy to mitigate breast cancer metastasis. Recent preclinical findings suggest that PCSK9 inhibition may, in fact, enhance the efficacy of HER2-targeted breast cancer therapies [46]. Additionally, PCSK9 inhibition has emerged as a promising strategy to enhance the efficacy of anti-PD-1/PD-L1 immunotherapy [38–40], addressing the challenge of low patient response rates in cancer treatment [16].

Given its prognostic significance, PCSK9 may represent a valuable biomarker for stratifying cancer patients based on survival risk [18]. Furthermore, PCSK9 inhibition, which is already an established therapeutic strategy in cardiovascular disease, may have potential applications in oncology. The ability of PCSK9 inhibitors to modulate immune surveillance and enhance MHC-I expression raises the possibility that these agents could be combined with immune checkpoint inhibitors to improve antitumor immune responses. However, the dual nature of PCSK9’s role in different cancers underscores the need for further mechanistic studies to delineate the precise molecular pathways involved.

Future research should focus on elucidating the functional consequences of PCSK9 expression in different tumor microenvironments [18, 20, 47], assessing the effects of PCSK9 inhibition on cancer progression, and determining the potential benefits of PCSK9-targeted therapies in specific patient subgroups. Expanding our understanding of PCSK9’s diverse roles in cancer biology will be critical for harnessing its potential as a therapeutic target in oncology [10]. Lifestyle factors such as diet, exercise, and weight management can influence PCSK9 expression [48–52]. Notably, many of these lifestyle factors are also associated with cancer survival outcomes. However, the direct impact of these factors on PCSK9 expression within tumors remains unclear and warrants further investigation in future studies.

In conclusion, our study identifies PCSK9 as a prognostic biomarker with distinct, cancer-type-specific survival associations. Its role in immune modulation, lipid metabolism, and aging-related pathways highlights its importance as a potential therapeutic target in oncology. Further research is needed to clarify the mechanistic basis of PCSK9’s effects in different cancers and to explore its potential integration into precision medicine approaches for cancer treatment.

## Data Availability

The datasets analyzed during the current study are publicly available. RNA sequencing data were obtained from The Cancer Genome Atlas (TCGA) via the Genomic Data Commons (https://portal.gdc.cancer.gov/). Microarray gene expression data were accessed from the Gene Expression Omnibus (GEO) repository (https://www.ncbi.nlm.nih.gov/geo/), including datasets such as GSE96058. All accession numbers and relevant dataset details are provided in the Methods section. Additional data supporting the findings of this study are available from the corresponding author upon reasonable request.
